# Cryptogenic non-cirrhotic HCC: Clinical, prognostic and immunologic aspects of an emerging HCC etiology

**DOI:** 10.1038/s41598-024-52884-w

**Published:** 2024-02-21

**Authors:** Boris J. B. Beudeker, Rael Guha, Kalina Stoyanova, Jan N. M. IJzermans, Robert A. de Man, Dave Sprengers, Andre Boonstra

**Affiliations:** 1 Department of Gastroenterology and Hepatology, Erasmus MC University Medical Center, Rotterdam, The Netherlands; 2 Department of Surgery, Erasmus MC University Medical Center, Wytemaweg 80, 3015 CN Rotterdam, Postbus 2040, 3000 CA Rotterdam, The Netherlands

**Keywords:** Risk factors, Predictive markers, Hepatocellular carcinoma

## Abstract

The incidence of hepatocellular carcinoma (HCC) in non-cirrhotic livers is rising significantly, but clear risk factors for screening remain elusive. This study sought to characterize non-cirrhotic HCC etiologies. HCC cases from 2009 to 2020 in a Dutch referral center were examined, revealing 371 out of 1654 cases (22%) as non-cirrhotic. Notably, the incidence of non-cirrhotic HCC increased by 61% in the time frame between 2009 and 2020. Interestingly 39% of non-cirrhotic HCC cases had cryptogenic origins. Cryptogenic non-cirrhotic HCC exhibited similarities with non-cirrhotic NAFLD HCC, but displayed advanced tumor stages, lower surgical rates, and a more frequent presence of symptoms, which substantiated in poor survival rates. Advanced cryptogenic non-cirrhotic HCC stages exhibited elevated serum interleukin-6 levels compared to non-cirrhotic HCC with defined etiologies. Comparative analysis encompassing cryptogenic and NAFLD non-cirrhotic HCC cohorts and controls unveiled comparable circulating immune biomarker profiles and PNPLA3 polymorphisms. To conclude, the primary etiology of non-cirrhotic HCC in our cohort has not defined risk factors. This cryptogenic variant exhibits distinct traits, such as advanced tumors and increased symptoms, and most resemble burned-out NAFLD. Understanding this HCC variant is crucial for improving screening and management strategies.

## Introduction

Hepatocellular carcinoma (HCC) is an important cause of cancer-related mortality worldwide^1^. Patients are often diagnosed with advanced disease and consequently have poor prognosis due to limited treatment options. HCC surveillance is crucial as it detects and allows treatment of disease early thereby, improving the chances of a cure. Screening typically includes biannual measurements of serum alpha-fetoprotein (AFP) levels and ultrasound examinations^2,3^. Globally, the most frequent risk factor for HCC is liver cirrhosis caused by chronic infections with hepatotropic viruses, i.e., hepatitis B virus (HBV) and hepatitis C virus (HCV), but also alcohol abuse, fatty liver disease, autoimmune disease and cryptogenic causes of cirrhosis are important risk factors^1,4,5^. In line with these risk factors, polymorphisms in important metabolic genes, such as patatin-like phospholipase domain-containing protein 3 [*PNPLA3*]) involved in the pathogenesis of NAFLD, have a reported 12-fold risk for HCC^6–9^. Since the majority of HCC develops in cirrhotic livers, much research has been focused on studying, screening and treating these individuals^10^. However, HCC also occurs in non-cirrhotic livers, especially in patients with non-alcohol fatty liver disease (NAFLD)^11,12^. Additionally, there is a lack of clearly defined risk factors that would justify screening this population. Interestingly, studies on HCC etiology fail to clearly define up to 48% of non-cirrhotic HCC cases^12–15^, a subset that we later identify in this study as cryptogenic non-cirrhotic HCC. The increasing incidence of NAFLD, as well as other important HCC risk factors, such as the metabolic syndrome: a collection of conditions including type II diabetes, obesity, dyslipidemia, and hypertension are an emerging cause of cirrhotic and non-cirrhotic HCC globally^1^.

HCC arises almost exclusively in the context of liver inflammation and is closely associated with altered levels of circulating immune mediators, such as the proinflammatory cytokine interleukin- (IL) 6^16^. In our pursuit of HCC screening biomarkers, we recently delved into circulating immune profiles, such as IFN-γ, interleukins, hepatocyte growth factor and important hormones, in cirrhotic HCC, uncovering a strong connection with underlying liver disease^4^. The question of whether similar associations exist for non-cirrhotic HCC etiologies remains unanswered. However, this information bears significance, as it holds the potential to enhance our comprehension of liver disease progression mechanisms and could pinpoint a subgroup that stands to gain from screening. With the increasing global prevalence of NAFLD and metabolic syndrome, it is crucial to undertake a thorough exploration of the characteristics of non-cirrhotic HCC. This entails a dedicated effort towards identifying HCC risk factors that can facilitate efficient screening measures. We aim to characterize patients with non-cirrhotic HCC and explore clinical risk factors, single nucleotide polymorphisms (SNPs), and circulating immune mediators.

## Results

### Incidence and characteristics of cryptogenic non-cirrhotic hepatocellular carcinoma

First, we aimed to investigate the incidence and clinical characteristics of HCC, with a focus on non-cirrhotic HCC, in Rotterdam, the Netherlands between 2009 and 2020.

**Figure 1:**
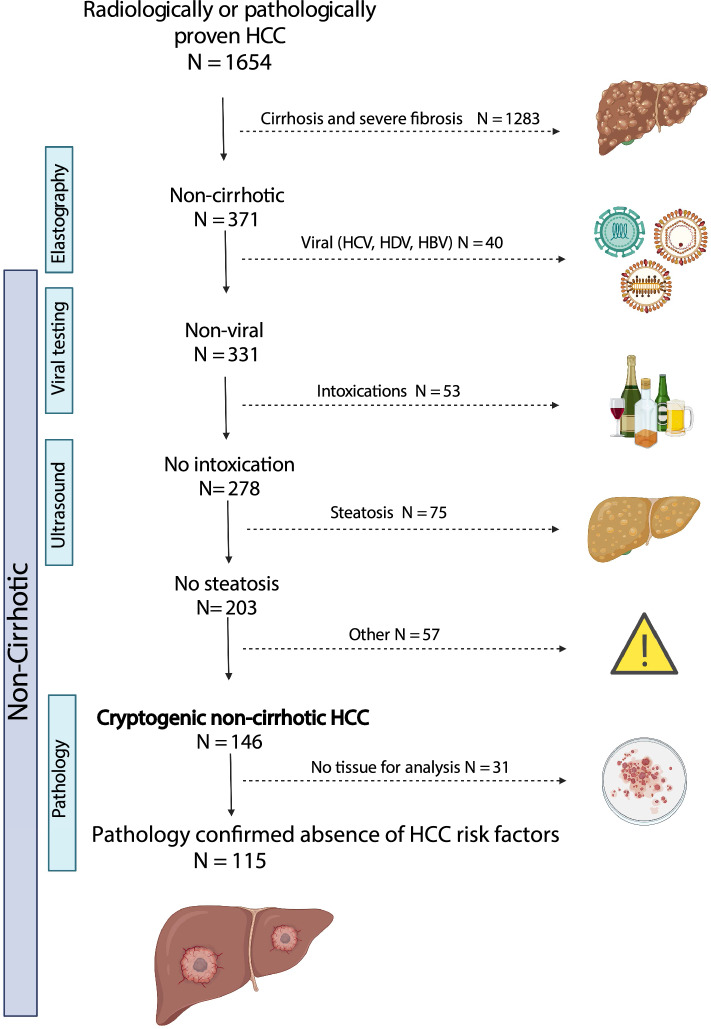
Schematic representation of categorization and identification of study patients.

We analyzed data from 2304 HCC cases and identified 1654 cases that met our specified criteria, as outlined in the “Methods” section. Among these cases, 371 (22%) were diagnosed with non-cirrhotic HCC (fibroscan < 7 kPa or metavir < F2), while the majority (78%) exhibited HCC in a background of severe fibrosis or cirrhosis (fibroscan > 12 kPa or metavir > F3). The determination of a non-cirrhotic background involved a combination of imaging and liver biopsy or pathology study of resection material in 127 (34%) and 67 (18%) cases, respectively, and 177 (48%) cases were identified using fibroscan and imaging alone. Cases with high certainty of non-cirrhotic HCC was not confirmed via biopsy. The most common etiologies with severe fibrosis or cirrhosis-associated HCC were ALD (32%), combined viral and non-viral causes (16%), and NAFLD (14%). In the non-cirrhotic HCC group, the majority were associated with non-viral risk factors such as ALD (14%), NAFLD (22%) or other non-viral causes (15%), such as HCC (3%), hemochromatosis (2%), porphyria (2%), cholestatic liver disease (1%). A minority were diagnosed with viral HCC associated with chronic HBV (6%), HCV (0.8%), or co-infection and combined viral and non-viral etiology (4%). A unique group of 146 (39%) patients had no significant degree of liver fibrosis on fibroscan (< 7 kPa), were negative for a history of hepatitis, had negative HBV and HCV serology, no history of autoimmune disease or cholestatic liver disease, had no history of significant alcohol use, and had no evidence of steatosis on MRI or ultrasound studies (Fig. 1). This group of patients was categorized as cryptogenic non-cirrhotic HCC. Among this group, 115 had pathology report with data on non-tumor parts of the liver. Specifically, 43 patients had data obtained from resection materials, 70 from liver biopsies and 2 from autopsies. The biopsies were indicated either to investigate the uncertain underlying etiology of the HCC, previously because of abnormal liver test or were acquired during tumor biopsy procedures. The pathological report confirmed the absence of HCC risk factors such as inflammation, significant fibrosis (F2), macrovesicular steatosis, iron overload, and hepatic ballooning in the liver tissues.

**Figure 2:**
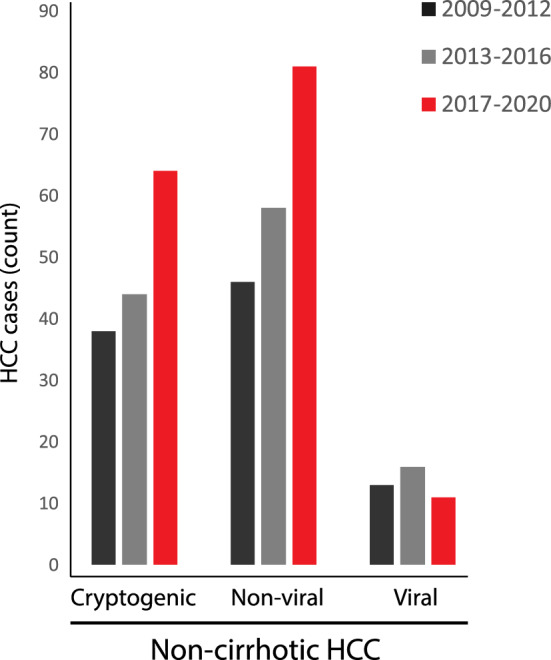
Non-cirrhotic HCC incidence of patients with cryptogenic, viral and non-viral HCC stratified by periods from 2009 to 2020.

Over a 12-year period, from 2009 to 2020, the number of patients diagnosed with non-cirrhotic HCC increased by 61%, largely due to an increase in the number of patients with cryptogenic and NAFLD-associated HCC (Fig. 2).

### Clinical characteristics of non-cirrhotic HCC patients with cryptogenic etiology compared to those with NAFLD, ALD, and HBV etiologies

Given the comparable rise in NAFLD and cryptogenic non-cirrhotic HCC, we next aimed to characterize these groups, in addition to the two second-largest non-cirrhotic cohorts, HBV and ALD. As shown in Table 1, we found that patients with cryptogenic HCC had larger tumors and a lower frequency of early-stage tumors compared to those with NAFLD and HBV etiologies. Notably, none of the patients diagnosed with cryptogenic and NAFLD-associated non-cirrhotic HCC had undergone any form of screening. Upon diagnosis, a higher percentage of cryptogenic and ALD patients reported symptoms and significant weight loss compared to those with NAFLD and HBV etiologies. Symptoms were often aspecific, and included abdominal pain, flank pain, radiating pain, bloating, palpable mass, fatigue, malaise, nausea, diarrhea, anorexia, weight loss and or fever.

**Table 1:** Clinical characteristics of patients with HCC in a non-cirrhotic liver stratified by underlying etiology.

		Cryptogenic	NAFLD	ALD	HBV	*p* value	NAFLD versus cryptogenic *p* value
N =		115	75	53	23		
Age	Median (IQR)	70 (18.5)	75 (27)	69 (9)	55 (27)	< 0.001	0.723
Sex							
	Female	38 (33%)	30 (40%)	7 (13%)	4 (17%)	0.005	0.328
	Male	77 (67%)	45 (60%)	46 (87%)	19 (83%)		
Ethnicity							
	African	1 (1%)	1 (1%)	1 (2%)	4 (17%)	< 0.001	0.841
	Asian	1 (1%)	0 (0%)	0 (0%)	11 (48%)		
	Caucasian	112 (97%)	73 (98%)	52 (98%)	6 (26%)		
	Other	1 (1%)	1 (1%)	0 (0%)	2 (9%)		
Smoking		61 (53%)	35 (47%)	35 (66%)	11 (49%)	0.203	
Excessive alcohol use		0 (0%)	0 (0%)	50 (94%)	1 (4%)	< 0.001	
Positive family history		10 (9%)	15 (20%)	3 (6%)	5 (22%)	0.012	0.017
Symptomatic		85 (74%)	40 (53%)	33 (62%)	14 (60%)	0.113	
Weight loss		36 (31%)	10 (13%)	15 (28%)	1 (4%)	0.003	0.005
BMI	Median (IQR)	25 (5)	28 (6)	26 (6)	24 (5.5)	< 0.001	< 0.001
Diabetes mellitus II		29 (25%)	38 (51%)	20 (38%)	5 (21%)	0.002	< 0.001
Hypertension		75 (65%)	54 (72%)	42 (79%)	9 (39%)	0.026	0.383
Dyslipidemia		31 (27%)	32 (43%)	31 (58%)	6 (26%)	0.001	0.016
Oral statins		14 (12%)	13 (17%)	2 (4%)	3 (13%)	0.143	
ALT (U/L)	Median (IQR)	44 (56)	37.5 (44)	47.5 (48)	32 (16)	0.227	
AST (U/L)	Median (IQR)	70 (89)	39.5 (33)	52 (98)	40 (49)	0.007	< 0.001
AFP (ng/mL)	Median (IQR)	34 (1632)	7 (304)	21.5 (1891)	99 (14810)	0.236	
FIB-4	Median (IQR)	2.6 (2.7)	1.8 (1.1)	1.7 (2.3)	2.1 (1.7)	0.042	0.005
APRI	Median (IQR)	0.62 (0.74)	0.41 (0.42)	0.49 (0.78)	0.44 (0.58)	0.261	
Fibrosis >F1		7 (6%)	9 (12%)	6 (11%)	2 (9%)	0.501	
HBV (anti-HBc pos)		0 (0%)	0 (0%)	0 (0%)	23 (100%)		
HCV		0 (0%)	0 (0%)	0 (0%)	0 (0%)		
Surveillance	Yes (%)	0 (0%)	0 (0%)	1 (2%)	12 (52%)	< 0.001	
Treatment strategy						< 0.001	<0.001
Curative		43 (40%)	52 (71%)	30 (57%)	12 (52%)		
Non-curative		23 (20%)	9 (12%)	10 (19%)	7 (30%)		
Best supportive		43 (40%)	13 (17%)	13 (25%)	4 (17%)		
Tumor stage							
	Early	18 (17%)	26 (36%)	15 (28%)	7 (32%)	0.033*	0.004
	Intermediate	61 (54%)	36 (49%)	26 (49%)	11 (50%)		
	Advanced	32 (29%)	11 (15%)	12 (23%)	4 (18%)		
Tumor size (mm)	Median (IQR)	110 (66)	65.5 (71)	100 (90)	74 (39)	< 0.001	< 0.001

Associations between variables were tested using an ANOVA, chi-square, or their nonparametric equivalents when appropriate. Next, only significant variables were tested for a difference between non-cirrhotic NAFLD HCC and non-cirrhotic cryptogenic HCC using student’s t test, chi-square, or their nonparametric equivalents when appropriate.

*p value for comparison early versus non-early.

Cryptogenic and NAFLD non-cirrhotic HCC patients were older, and had a more balanced gender ratio, while ALD and HBV were more commonly diagnosed in men. Cryptogenic, ALD, and NAFLD patients were predominantly Caucasian, while HBV was more frequently found in Asian and African individuals. BMI at diagnosis was highest in NAFLD, followed by ALD, cryptogenic and HBV. NAFLD and ALD had the highest rates of diabetes, hypertension, and dyslipidemia, while these were less frequent in cryptogenic and HBV non-cirrhotic HCC patients. No significant differences were found in the prevalence of smoking or the use of oral statins between the groups.

In summary, both NAFLD and cryptogenic cases shared similar characteristics, including age, sex, ethnicity, smoking habits, alcohol use, hypertension, and oral statin use. However, notable differences emerged regarding rate of symptoms, type 2 diabetes, weight loss, BMI, and dyslipidemia. Recognizing the potential impact of advanced tumor stage and weight loss on these factors, our analysis focused on non-early stages of non-cirrhotic cryptogenic and NAFLD HCC. Compared to patients with NAFLD-associated HCC, those with cryptogenic HCC were more likely to experience symptoms (77% vs. 57%; *p* = 0.038). Additionally, they reported a higher prevalence of significant weight loss (> 10% total body weight) at the time of diagnosis (34% vs. 17%; *p* = 0.035) and were less likely to undergo surgical treatment (33% vs. 64%; *p* = 0.015).

### Tumor size and the presence of non-cirrhotic HCC exert notable impacts on serum markers PIVKA-II, IL-6 and the FIB-4 fibrosis score

Serum ALT levels and serum AFP levels were comparable across the four groups, while cryptogenic non-cirrhotic HCC patients had higher levels of AST. Two other tumor markers were evaluated: CA19.9 and PIVKA-II (also known as DCP). As compared to NAFLD-associated patients, higher levels of serum PIVKA-II were found in cryptogenic non-cirrhotic HCC (log 3.3 (IQR 4.9) vs. log 4.1 (IQR 4.2; *p* = 0.002), which was partly explained by the larger tumors in cryptogenic etiology (tumor size and PIVKA-II; *r* = 0.34, *p* < 0.001). As expected, levels of the cholangiocarcinoma marker CA19.9 were comparable between both groups. The results of our study showed that the FIB-4 fibrosis score, based on AST, ALT, and platelet levels, was significantly higher in the cryptogenic group compared to the NAFLD group (Table 1). The majority of non-cirrhotic HCC patients with pathology-proven Metavir F0 or F1 had a FIB-4 value of > 1.3 while patients with Metavir > F2 had a FIB-4 value of < 1.3 (Fig. S2). This resulted in a specificity of 11% and sensitivity of 70% for the FIB-4 score in predicting fibrosis in non-cirrhotic HCC patients.

### In a multivariable Cox regression analysis, treatment strategy, tumor stage and age jointly explain the overall survival of non-cirrhotic HCC

**Figure 3:**
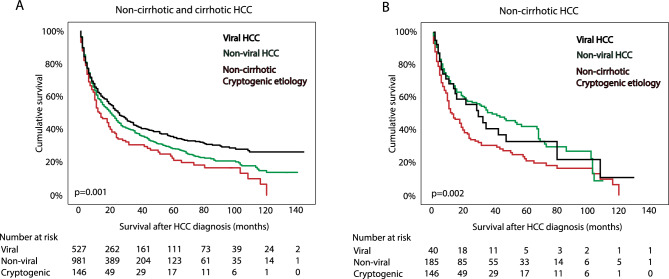
Survival proportion after the diagnosis of de novo hepatocellular carcinoma (HCC) in patients with viral hepatitis-related HCC in cirrhotic or non-cirrhotic liver, non-viral related HCC in cirrhotic and non-cirrhotic and in those with cryptogenic HCC. Regardless of an absence of HCC risk factors, cryptogenic HCC is linked with worst survival than those with viral or non-viral related HCC causes. The cumulative survival was calculated with the univariate cox regression model.

Next we sought to explore how non-cirrhotic cryptogenic etiology impacts overall survival. The first step involved examining the relationship between liver disease etiology, tumor stage, and overall survival in a cohort of 1654 HCC patients, which included both cirrhotic and non-cirrhotic cases (Table 2). In univariate analysis, non-cirrhotic cryptogenic etiology was associated with higher mortality as compared to distinct non-cirrhotic and cirrhotic HCC etiologies (log rank *p* = 0.001 and *p* = 0.002) (Fig. 3A,B). Other factors that were found to be associated with survival in univariate analysis were age, severe fibrosis (F3–F4) and tumor stage, while diagnostic period and sex were not. In a multivariable analysis, cryptogenic non-cirrhotic etiology was found to be an independent negative prognostic factor as compared to viral and non-viral etiology (*p* = 0.037), along with intermediate and advanced tumor stage and older age. While treatment strategy in cirrhotic HCC is guided by the Barcelona Clinic Liver Cancer guidelines^17^, our analysis of non-cirrhotic HCC demonstrated that curative treatment was also offered in intermediate and advanced HCC cases (see Table 1 and data not shown). Subsequently, we conducted a multivariable analysis in non-cirrhotic HCC, incorporating treatment strategy (Table 3). The results indicated that survival in the non-cirrhotic group was significantly influenced by age, tumor stage, and treatment strategy. Notably, etiology no longer played a significant role in predicting survival. Moreover, intermediate tumor stage did not emerge as a predictor for survival in the non-cirrhotic group (*p* = 0.79).

**Table 2:** Factors associated with overall survival in univariate and multivariate analyses of cirrhotic and non-cirrhotic HCC (N = 1654).

Parameters	Univariate analysis			Multivariate analysis		
	HR	95% CI	*p* value	HR	95% CI	*p* value
Age	1.017	1.011–1.023	< 0.0001	1.008	1.002–1.014	0.009
Sex (male)	0.916	0.793–1.058	0.234			
Severe fibrosis (F3–F4)	1.034	0.893–1.197	0.658	1.398	1.144–1.681	< 0.0001
Etiology			0.001			0.037
Cryptogenic	Reference			Reference		
Non-viral	0.808	0.657–0.995		0.783	0.603–1.017	
Viral	0.677	0.543–0.845		0.696	0.522–0.927	
Tumor stage			< 0.0001			< 0.0001
Early stage	Reference			Reference		
Intermediate stage	2.113	1.785–2.501		2.159	1.821–2.560	
Advanced stage	6.662	5.703–7.784		6.606	5.642–7.734	
Diagnostic period			0.429			
2009–2012	Reference					
2013–2016	0.906	0.782–1.051				
2017–2020	0.947	0.810–1.107				

*HR* hazard ratio, *CI* confidence interval.

**Table 3:** Factors associated with overall survival in univariate and multivariate analyses of non-cirrhotic HCC (N = 371).

Parameters	Univariate analysis			Multivariate analysis		
	HR	95% CI	*p* value	HR	95% CI	*p* value
Age	1.021	1.010–1.031	< 0.0001	1.015	1.004–1.025	0.006
Sex (male)	0.809	0.606–1.081	0.153			
Etiology			0.004			0.440
Cryptogenic	Reference			Reference		
Non-viral	0.628	0.477–0.827		0.843	0.636–1.119	
Viral	0.688	0.441–1.074		0.825	0.523–1.301	
Tumor stage			< 0.0001			0.003
Early stage	Reference			Reference		
Intermediate stage	1.313	0.910–1.894		1.054	0.705–1.574	
Advanced stage	4.966	3.530–6.986		2.203	1.292–3.755	
Treatment strategy			< 0.0001			< 0.0001
Curative	Reference			Reference		
Non-curative	3.679	2.627–5.151		2.349	1.505–3.665	
Best supportive	5.583	4.037–7.721		2.524	1.496–4.260	
Diagnostic period			0.883			
2009–2012	Reference					
2013–2016	1.053	0.759–1.460				
2017–2020	0.947	0.775–1.533				

*HR* hazard ratio, *CI* confidence interval.

### Cryptogenic and NAFLD non-cirrhotic HCC have comparable circulating immune profiles and risk SNPs

To further explore the non-cirrhotic cryptogenic etiology, single nucleotide polymorphisms (SNPs) associated with increased risk for NAFLD and HCC were determined. Supplementary Table 2 shows the prevalence of the risk allele (G) of SNP *PNPLA3* and (T) of SNP *MBOAT7* in Caucasian patients. The results did not show a significant risk profile for liver disease in the cryptogenic and NAFLD non-cirrhotic HCC groups compared to healthy controls.

As we demonstrated an increase in AST levels in non-cirrhotic cryptogenic HCC, we further assessed serum levels of CRP and IL-6 as proxies for immune involvement in this specific subset of non-cirrhotic HCC. While CRP levels were not associated with tumor size or etiology, higher levels of IL-6 were observed in the cryptogenic compared to the NAFLD non-cirrhotic HCC group (*p* < 0.001) (Fig. S3). Among cryptogenic cases, larger tumors were positively associated with IL-6 (r = 0.29, *p* < 0.001). Liver disease etiologies are strongly associated with circulating cytokines^3^, therefore a more extensive evaluation of 28 circulating cytokines was conducted in a sub-cohort of 30 cryptogenic non-cirrhotic HCC cases, 28 NAFLD non-cirrhotic HCC cases, and 18 NAFLD controls without HCC (Table S1). Among the cytokines were various chemokines (i.e., CXCL12, CXCL8), growth factors (i.e., G-CSF, HGF), and interleukins. A modest trend in pentraxin-3 levels was found between the cryptogenic and NAFLD groups (Fig. S4A). No differences were observed in the key inflammatory cytokines IFN-y and TNF, or in the levels of anti-inflammatory cytokines, such as IL-10 and IL-1ra (Fig. S4B). When comparing the cryptogenic non-cirrhotic HCC group to the NAFLD control group, serum levels of IL-8 (CXCL8) were significantly increased in cryptogenic non-cirrhotic HCC (Fig. S4C). In this sub-cohort with comparable tumor stages, IL-6 was no longer found to be significantly different.

## Discussion

NAFLD non-cirrhotic HCC is an emerging cause of HCC, and there is a lack of risk factors and screening biomarkers to identify important subgroups eligible for screening. This study aimed to identify and characterize risk factors of non-cirrhotic HCC. In our retrospective analysis of 1654 HCC cases, we observed that 22% of cases manifested in non-cirrhotic livers (fibroscan < 7 kPa), with 39% of these cases attributed to cryptogenic origin. Previous studies have reported indeterminate causes in up to 48% of non-cirrhotic HCC cases, raising concerns about potential misclassification due to incomplete data hindering definitive determinations^5,11,13–15,18,19^. Among the studies on cryptogenic causes of HCC, a South Korean investigation reported the lowest rate (< 8%) of cryptogenic HCC (cirrhotic and non-cirrhotic), while Dutch, United States, and Turkish studies documented frequencies ranging between 11 and 19%^12,13,20,21^. Our study firmly establishes cryptogenic etiology as the dominant underlying liver condition in non-cirrhotic HCC patients within our cohort. Notably, pathological assessments validated the lack of significant risk factors within this subgroup.

Our research highlights the growing prevalence of non-cirrhotic HCC, mainly due to the increasing occurrence of non-cirrhotic NAFLD and non-cirrhotic cryptogenic etiology. In line with a previous analysis in our center, only a small number of non-cirrhotic cases were attributed to HCV^12^. Although non-cirrhotic cryptogenic HCC stands out as a distinct subgroup within non-cirrhotic HCC, characterized by more advanced tumor stage, higher rates of symptomatic disease, increased instances of weight loss before diagnosis, and a lower rate of curative treatment, it shares notable similarities with NAFLD non-cirrhotic patients in terms of age, sex, ethnicity, smoking habits, alcohol use, hypertension, medication use. Differences in lipid levels, diabetes, and BMI between non-cirrhotic NAFLD HCC and cryptogenic HCC could potentially be explained by tumor stage-associated anorexia and weight loss^22–26^, and therefore cryptogenic HCC may represent a subgroup with burned-out NAFLD. Multivariable analysis of non-cirrhotic HCC pointed out that the generally poor survival of cryptogenic etiology was explained by tumor stage and treatment strategy and not by etiology, which aligns with our findings of lower rate of curative treatment in the non-cirrhotic cohort. Due to the lack of curative treatment in a significant portion of patients with an unfavorable prognosis, detailed examination of histological factors was not conducted, as we have previously addressed this aspect^19^.

While we favor the hypothesis of burned-out NAFLD, it's important to acknowledge the potential role of other factors like toxic exposures (including misrepresentation of alcohol use), metabolic risk factors, genetic influences, and the impact of the gut microbiome^27–29^.

Survival outcomes in the univariate analysis of non-cirrhotic HCC were comparable to those of HCCs arising in a cirrhotic liver, consistent with prior research findings^12,20,30,31^. The non-cirrhotic cryptogenic group presented with the worst survival as compared to other groups given they were detected generally late and with advanced and symptomatic disease. In line with a previous report^32^, non-cirrhotic HCC cases were more likely to receive curative therapy, and these groups may benefit from more aggressive therapies compared to cirrhotic HCC. Still, early detection is a goal, however, clear subgroups that could benefit from screening are not clear yet.

In our efforts to better understand non-cirrhotic cryptogenic HCC, various biomarkers were evaluated. Serum AFP did not exhibit significant differences across the various etiologies, consistent with findings from prior research^33^. We found higher serum levels of both PIVKA and IL-6 in non-cirrhotic cryptogenic HCC compared to other HCC causes. PIVKA-II and IL-6 levels are likely linked to tumor stage, as they correlated with tumor size. In support of this, when we matched tumor stages in the NAFLD and cryptogenic subgroups, this significant association for both markers was no longer evident. The correlation between IL-6 levels and tumor size may indicate a possible role in signaling larger immunologically active tumors^16^. Of the 28 cytokines in our subgroup analysis, only pentraxin-3 and IL-8 showed a discernible trend. Although increasing the sample size might influence statistical significance, the study’s focus was on more substantial differences that could have a greater impact on our conclusions. We previously showed that circulating cytokines show a strong association with underlying liver disease. The similarities in cytokine levels in NALFD and cryptogenic HCC may therefore point towards a similar origin of their HCC, such as NAFLD. A recent European study identified MIG and MIF^34^ as biomarkers for distinguishing non-cirrhotic NAFLD-associated HCC from NAFLD controls. Consistent with our previous findings in a larger cohort, these markers held no value in the detection of NAFLD-related complications. The FIB-4 score, commonly used to assess liver fibrosis at a value greater than 1.3^2^, showed limited sensitivity in our study and is likely biased by HCC the impact of HCC on AST levels and its use should therefore be used with caution in non-cirrhotic HCC.

We must acknowledge limitations in our study. Our study employed a comprehensive approach, integrating clinical chart review, radiology, pathology reports and laboratory findings for accurate liver disease diagnosis and tumor staging, excluding incomplete records. While potential biases from case complexity and documentation variations existed, we implemented measures to address them, enhancing the reliability of our data, especially the analysis of non-cirrhotic HCC cases (“Methods” section). To strengthen our findings, further investigation into factors such as histological tumor features, disease aggressiveness, timely diagnosis, causes of death, and surgical treatment constraints is warranted. Prospective cohort studies are necessary to conclusively validate our results.

Another limitation may be the inclusion of fibrosis stages up to one year before diagnosis. This factor did not apply to non-cirrhotic cryptogenic HCC or those with potentially curative disease, as their diagnostic workup inherently included fibrosis assessment, crucial for determining the appropriate therapy or upon pathology study of the liver tissue. The precision of our survival analysis was constrained by the absence of specific data on causes of death, relying on administrative records. Nevertheless, it's important to note that in non-cirrhotic HCC patients, liver decompensation has a lesser impact on mortality compared to patients with cirrhosis.

With the rising prevalence of NAFLD and non-cirrhotic HCC, future studies must explore this issue more comprehensively. Considering the diagnostic uncertainties associated with non-cirrhotic HCC, obtaining pathology confirmation is crucial not only to exclude other malignant causes but also to distinguish between HCC subgroups. Additionally, conducting immune histological studies on non-cirrhotic HCC may reveal potential immunological targets.

In conclusion, our study emphasizes the increasing occurrence of non-cirrhotic HCC associated with NAFLD and cryptogenic etiology. Non-cirrhotic cryptogenic HCC is connected with poor survival rates given they present with more advanced stages and limited treatment options, and on the basis of extensive prior weight loss, signify a form of burned-out NAFLD. With the significant mortality rates and the lack of specific screening guidelines for non-cirrhotic HCC, it becomes imperative to better understand the risk factors and improve quality of clinical management of this severe manifestation of HCC.

## Methods

### Patient identification and characterization

Patients with a primary diagnosis of hepatocellular carcinoma (HCC) at the Erasmus Medical Center between 2009 and 2020 were included in our study. The selection of patients was consecutive, ensuring a representative sample. Patient data were obtained from multiple sources, including the Netherlands Cancer Registry and hospital databases. The diagnosis of HCC was established based on histological and/or radiographic evidence, following the European guideline criteria^3^. Survival data were collected from both medical records and the Municipal Personal Records Database, which is a national database that is required to register all deaths. Exposure to HCC risk factors was collected from electronic patient records, including demographics, anamnestic data, laboratory results, and medical history. The etiology, tumor stage, and fibrosis levels were determined through physician documentation, imaging, pathology, and laboratory tests. Fibrosis stage that existed up to one year prior to the diagnosis were included in the analysis. The Barcelona Clinic Liver Cancer staging system was used for patients with severe fibrosis and cirrhotic HCC^17^, and since BCLC staging cannot be used for non-cirrhotic HCC a modified version of the eighth TNM edition was used. Fibroscan less than 7.0 kPa or Metavir less than F2 was considered non-cirrhotic. Severe fibrosis or cirrhosis was defined as fibroscan higher than 12.0 kPa, taking into account the liver disease etiology^2^, or Metavir scores of F3 and higher.

For non-cirrhotic HCC cases, the assigned hepatologists made the initial diagnosis, which was further verified by examining data fibroscan or pathology data on fibrosis, hepatic steatosis, medical history, alcohol use, viral serology, and tumor stage. For cryptogenic HCC cases in the non-cirrhotic group, a detailed medical history analysis involved assessing past illnesses, medication use, and potential alcohol and drug use. A more detailed “Methods” section can be found in the supplementary material.

### Ethics approval statement and patient consent statement

All patient samples and data used in this study were collected in the context of routine clinical patient care and the Institutional Review Board of the Erasmus Medical Center in Rotterdam approved of the use of these data and samples (METC-2017-1140 and MEC-2020-0383).

### Supplementary Information

Supplementary Information.

## Data Availability

The data supporting our findings are available from the authors upon reasonable request.
