# Supplementary material for: Cryptogenic non-cirrhotic HCC: Clinical, prognostic and immunologic aspects of an emerging HCC etiology
Source: Sci Rep. 2024 Feb 21;14:4302. doi: 10.1038/s41598-024-52884-w (PMC10881579; doi:10.1038/s41598-024-52884-w)
Supplement: Supplementary file 1 — Supplementary Information. [file 41598_2024_52884_MOESM1_ESM.pdf]

Supplementary file.

Cryptogenic non-cirrhotic HCC: Clinical, prognostic and immunologic aspects of an emerging HCC etiology

Boris J.B. Beudeker[1], Rael Guha[1], Kalina Stoyanova[1], Jan N.M. IJzermans[2], Robert A. de Man[1], Dave Sprengers[1], Andre Boonstra[1]

[1]Erasmus MC University Medical Center, Department of Gastroenterology and Hepatology, Rotterdam, the Netherlands

[2]Erasmus MC University Medical Center, Department of Surgery, the Netherlands

Corresponding author: Andre Boonstra, p.a.boonstra@erasmusmc.nl, Wytemaweg 80, 3015 CN Rotterdam, postbus 2040, 3000 CA Rotterdam

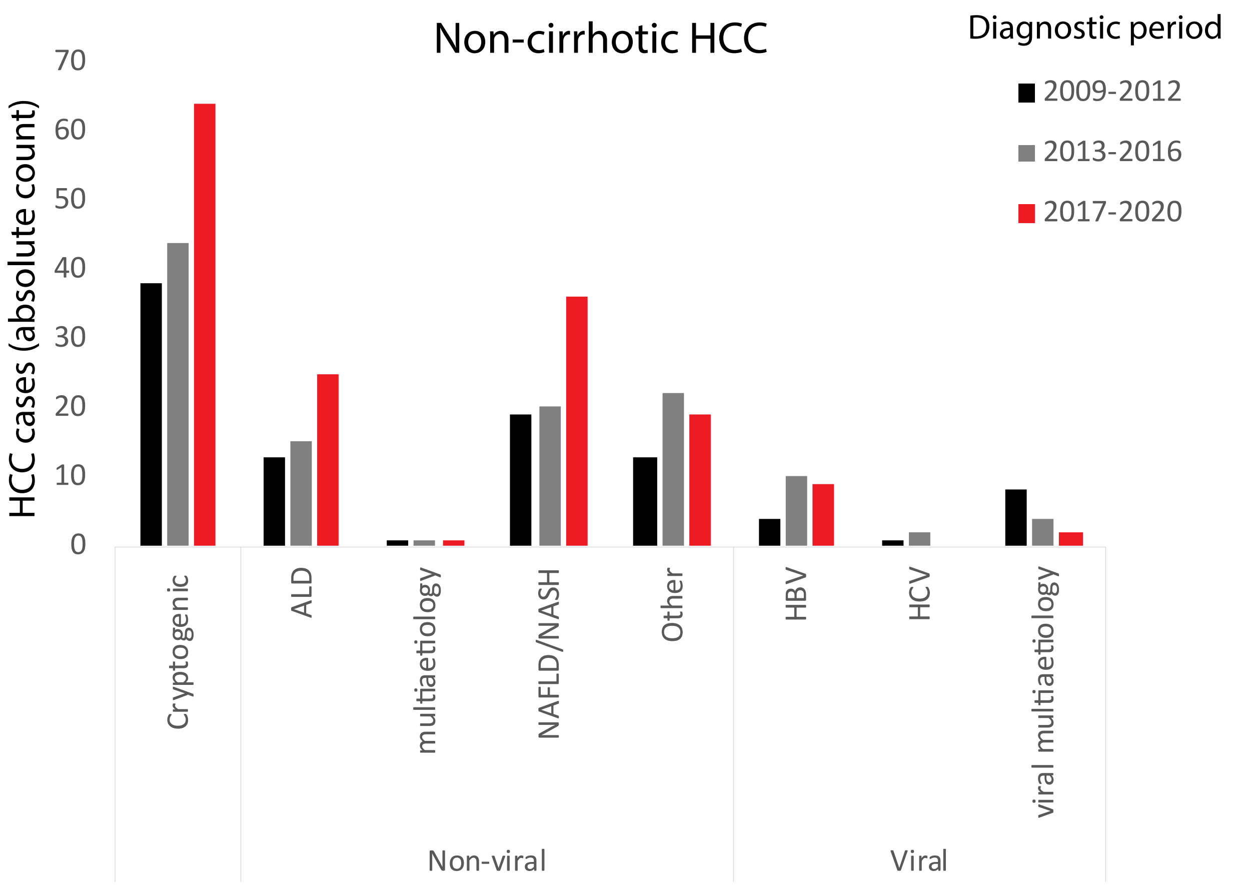

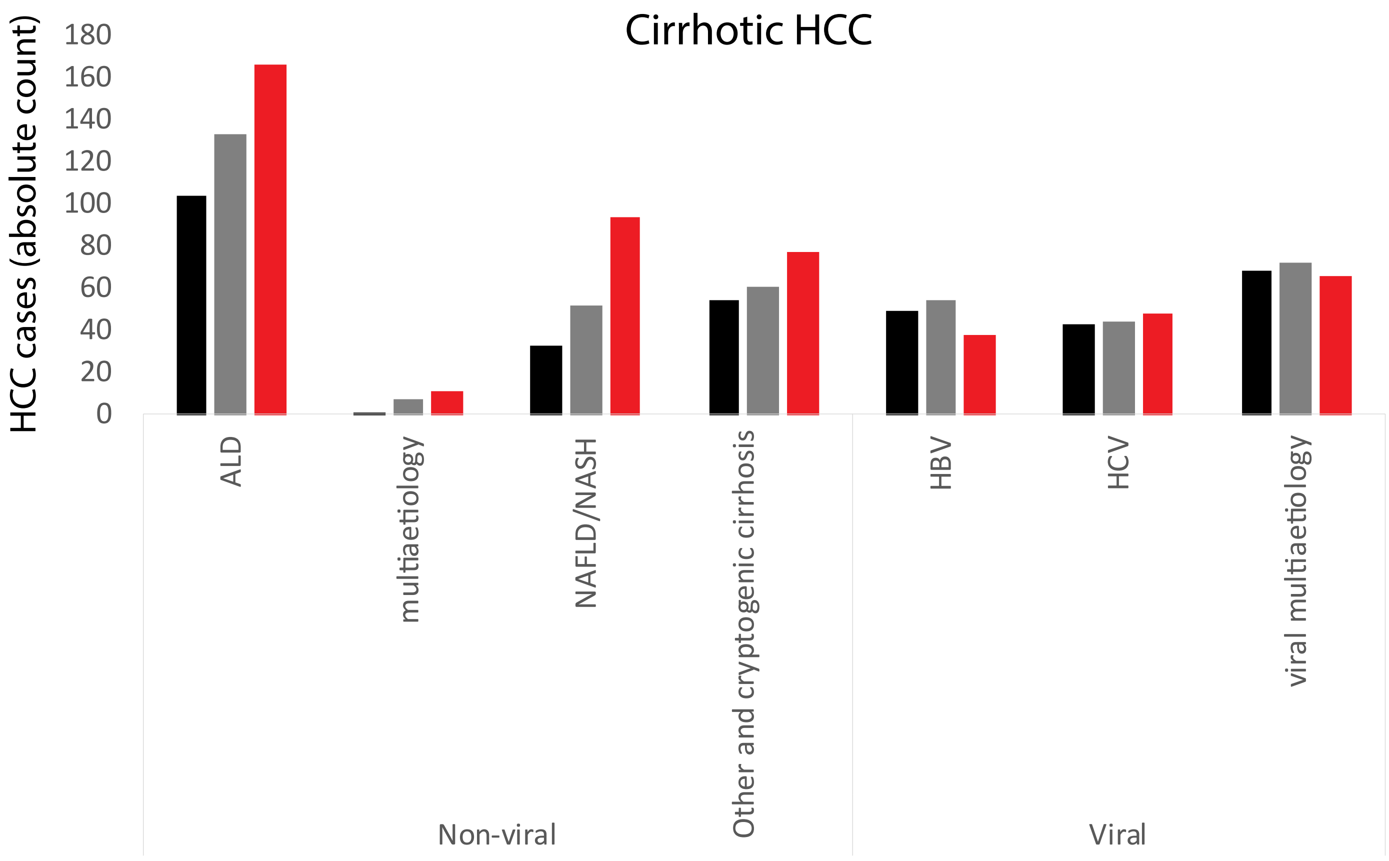

Supplementary figure 1. HCC incidence of patients with cirrhotic and non-cirrhotic HCC stratified by periods from 2009 to 2020 and underlying liver disease. Counts are absolute counts.

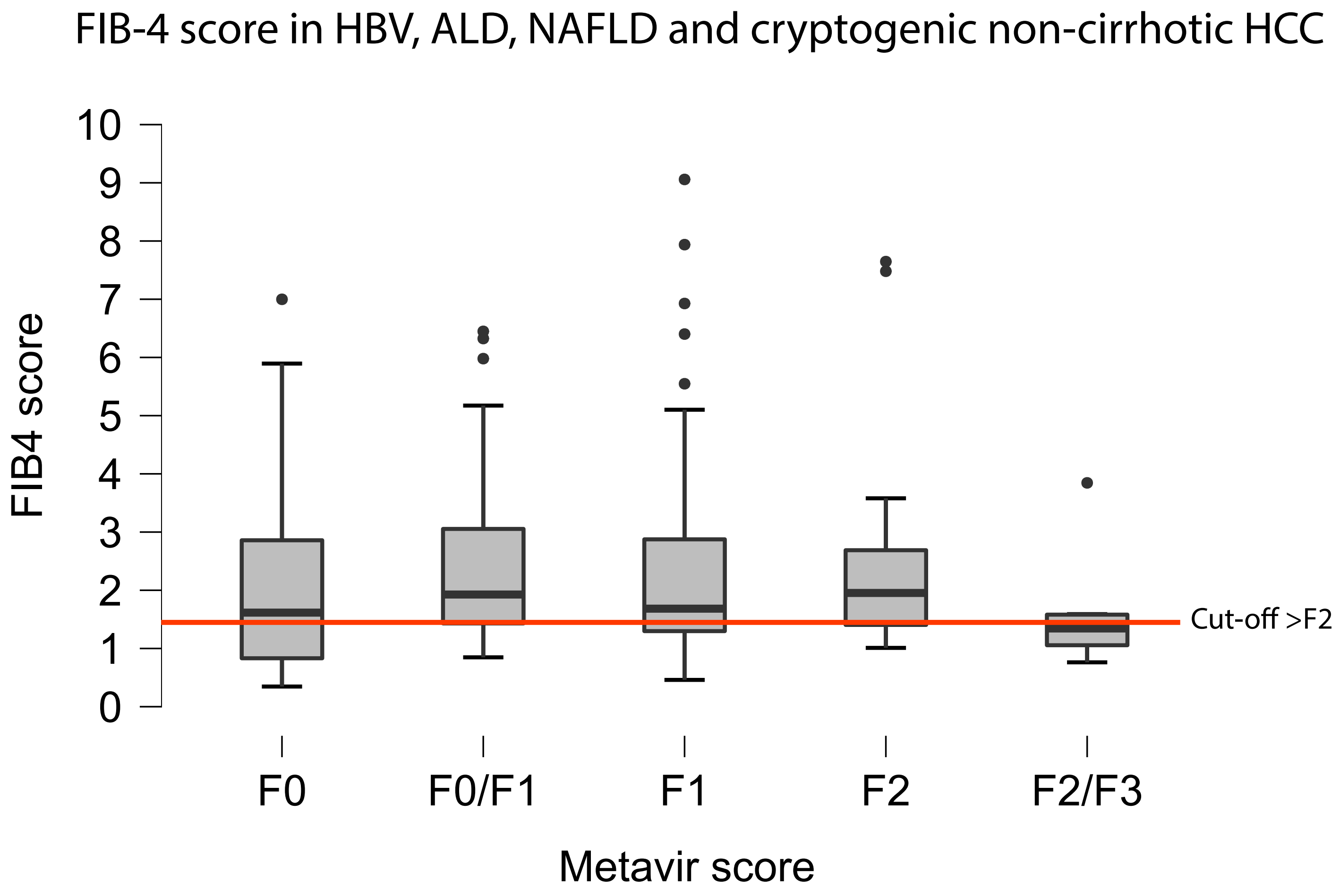

Supplementary figure 2.
The red line represents the FIB-4 cut-off value, proposed by the EASL guidelines for >F2 fibrosis

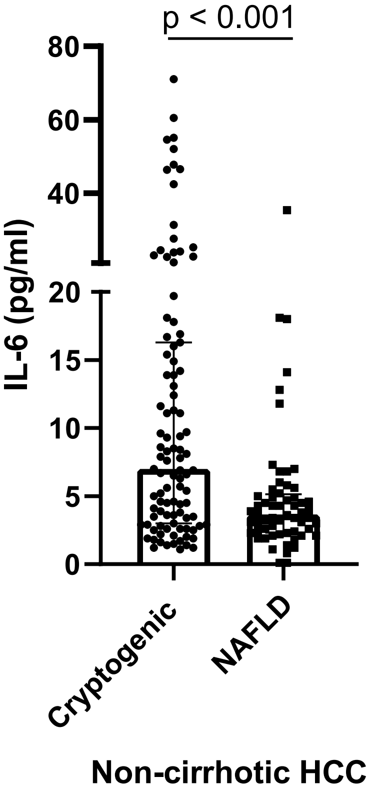

Supplementary figure 3.
Scatter dot plots with circulating interleuking-6 levels in non-cirrhotic cryptogenic HCC and non-cirrhotic NAFLD HCC.

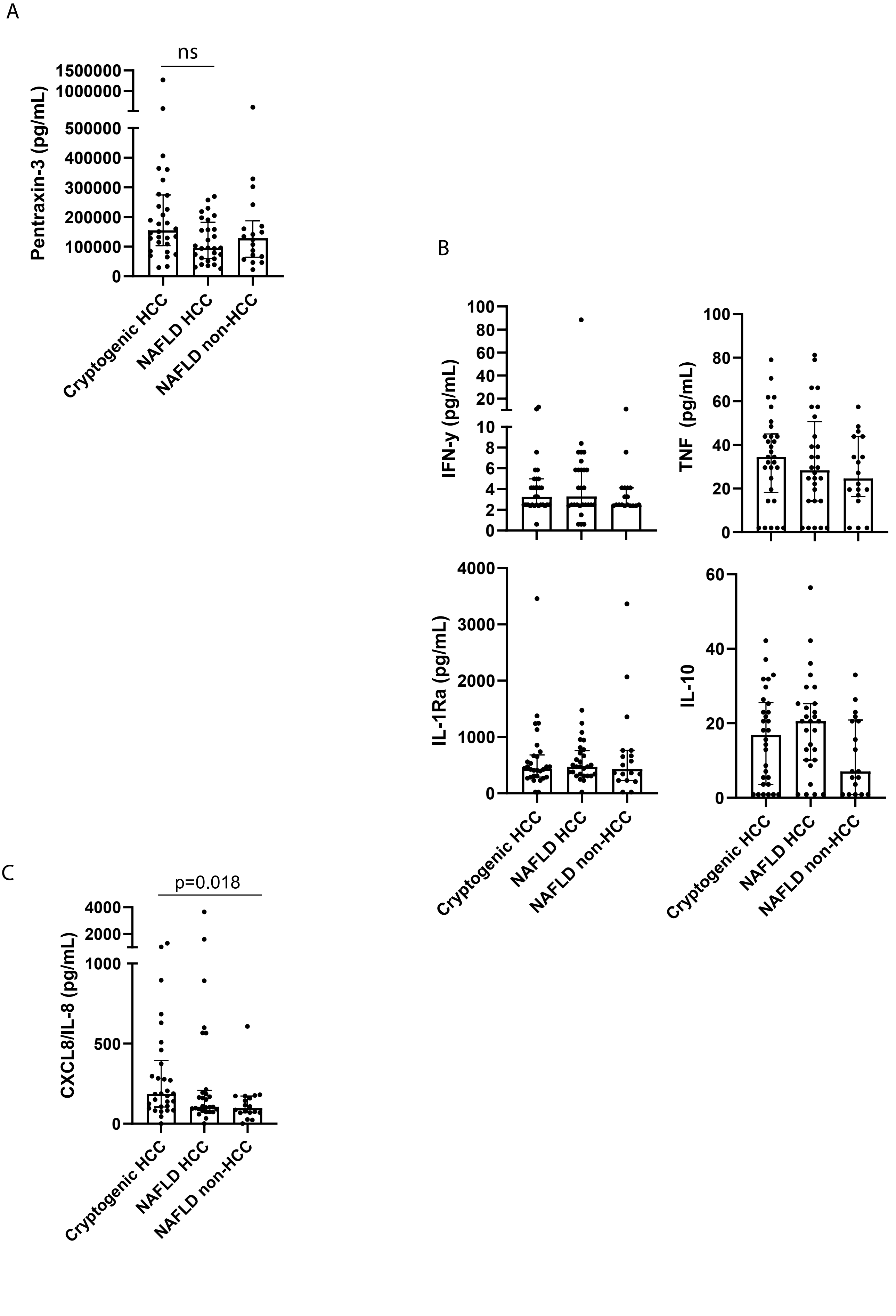

Supplementary figure 4.
Scatter dot plots with a selection circulating cytokines levels in non-cirrhotic cryptogenic HCC, non-cirrhotic NAFLD HCC and non-cirrhotic NAFLD controls: Pentraxin (A), IFN-γ, TNF, IL-10 and IL-1Ra (B) and CXCL8/IL-8 (C). All presented p-values are adjusted for multiple comparisons using the Bonferroni method.

Supplementary methods

Identification and characterization of study subjects

Patients with diagnosed HCC at the Erasmus Medical Center in the period 2009-2020 were identified. Data regarding incidence of HCC were extracted from the database of the Netherlands Cancer Registry, the automated pathological archive, Pathologisch-Anatomisch Landelijk Geautomatiseerd Archief (PALGA), and the National Registry of Hospital Discharge Diagnoses. The diagnosis of HCC required histological and/or radiographic evidence in accordance with the European guideline[3]. Data on survival after HCC diagnosis was obtained by screening the patient medical records and annual linkage to the Municipal Personal Records Database. Follow-up time was calculated from the date of diagnosis until the date of death, lost to follow-up or end of follow-up on January 1, 2022.

The data on exposure to HCC risk factors was collected from various sources, including the administrative registry of outpatient clinics, referral letters, scanned documentation from referring hospitals, and previous hospital admissions. These electronic records covered the time period of the current study, and written registries were also included for analysi s of risk factors prior to the implementation of the electronic system. The electronic patient records were searched for demographics, such as age at diagnosis, sex, ethnicity, body mass index (BMI), anamnestic data on symptoms, family history, medication use, medical history, smoking and alcohol consumption, blood pressure, laboratory results, including aspartate aminotransferase (AST), alanine aminotransferase (ALT), lipid levels, alpha-fetoprotein (AFP) and hepatitis virus serology. Smoking was defined as someone who regularly smoked (at least 1 cigarette per day) in the past 10 years or history of smoking with >20 pack years and "family history" was defined as reported data on family members with a history of HCC.

Assessment of etiology, tumor stage, and fibrosis:

To determine the etiology, tumor stage, and fibrosis levels, physicians' documentation and confirmatory imaging, pathology, and laboratory tests were used. Patients with viral etiology were tested for HBV, HCV, HCV/HBV or HBV/hepatitis D virus (HDV) co-infection. For non-viral causes of HCC, patients with alcoholic liver disease (ALD) related HCC had an estimated daily ethanol intake of more than >20 grams per day in females and >30 grams per day in males together with the presence of clinical and/or biological abnormalities suggestive of liver injury.

Patients with NAFLD-related HCC had had evidence of hepatic steatosis by histopathology, MRI or ultrasound, in the absence of alternative liver diseases. Other etiologies included acquired immune deficiencies, autoimmune liver disease, hemochromatosis, Wilson's disease, alpha-1 antitrypsin deficiency, primary biliary cholangitis, primary sclerosing cholangitis, a documented clinical history of immunomodulatory drugs, pathology-proven Metavir F2-F3 fibrosis without risk factors (significant fibrosis), and monogenic syndromes predisposing to HCC.

Tumor stage evaluation:
For patients with severe fibrosis and cirrhotic HCC, the Barcelona Clinic Liver Cancer (BCLC) staging system was used[3], categorizing the disease as early-stage (BCLC stage 0 and A), intermediate-stage (BCLC stage B), and advanced-stage (BCLC stage C and D). For non-cirrhotic HCC, a modified version of the eighth TNM edition was used, categorizing the disease as early-stage (TNM stage T1-T2, eligible for surgical treatment), intermediate-stage (TNM stage T3, eligible for a treatment), and advanced-stage (T4 or not eligible for treatment due to poor WHO performance status).

Fibrosis stage assessment:
In order to determine the fibrosis stage of the patients, we considered either the stage at the time of HCC diagnosis or the stage that existed up to one year prior to the diagnosis. The presence of severe fibrosis or cirrhosis was established either by the managing hepatologists or through pathology or liver transient fibroscan studies. Metavir F3 and higher were considered severe fibrosis or cirrhosis, while fibroscan less than 7.0 kPa or Metavir less than F2 was considered non-cirrhotic.

Inclusion and Exclusion Criteria:
Patients diagnosed with pathology or radiology-proven HCC[3] and having sufficient information on liver disease etiology, survival, tumor stage, and fibrosis level were included in the study. Etiology of HCC in cases with severe fibrosis and cirrhosis was established through a combination of methods, including: The assigned hepatologists' or liver transplantation team's diagnosis; thorough review of the patients' medical charts, including data on fibrosis, hepatic steatosis, medical history, alcohol use, viral serology, and tumor stage.

For non-cirrhotic HCC, the etiology was established through the assigned hepatologists' diagnosis, which was verified through a review of data on fibrosis, hepatic steatosis, medical history, alcohol use, viral serology, and tumor stage. The aim was to achieve a more accurate and comprehensive understanding of the etiology of HCC in patients with non-cirrhotic HCC. For cryptogenic non-cirrhotic HCC, the assigned hepatologists' diagnosis needed to be confirmed through review of liver ultrasonography or pathological study of non-HCC liver tissue, serum testing for hepatitis viruses, and a complete medical history, including past illnesses, medication use, and potential alcohol and drug use. Moreover, patients were excluded from the study in case of: HCC recurrence, mixed-type HCC, non-HCC liver metastases, age less than 18 years, co-existing non-HCC malignancies, residency abroad at the time of diagnosis, we excluded non-cirrhotic livers with fibroscan readings between 7 and 12 kPa for which no liver tissue was available to grade their fibrosis level

Research practices

The study employed a comprehensive approach by utilizing administrative data, laboratory findings, and clinical chart review to ensure a thorough collection of data. The etiology of cryptogenic cases was verified by a team of researchers working in collaboration with a gastroenterologist. It is important to acknowledge that the complexity of cases, variations in documentation practices among treating physicians, and the observational nature of our study may introduce potential biases into our results. To minimize these biases non-cirrhotic HCC cases underwent thorough scrutiny and accuracy of liver disease diagnosis and tumor stage was confirmed through examination of laboratory results, and imaging or pathology studies.

Assay methods

Circulating cytokines were measured in serum using the Bio-Plex platform, specifically our customized Bio-Plex human cytokine 28-plex panel (Bio-Rad Laboratories). In total, 28 experimental analytes were tested in three different panels: CCL13, CCL25, CCL3, CCL4, CCL5, CXCL1, CXCL11 CXCL12, CXCL13, CXCL2, CXCL8, G-CSF, HGF, IFN-y, IL-10, IL-16, IL-1Ra, IL-4, IL-6, MIF, MIG, MMP2, PDGF-BB, Pentraxin-3, SCF, TNF-a, TNF-b, TRAIL. In addition, AFP (ng/ml), PIVKA-II (AU/mL) and IL-6 (pg/mL) were measured (Fujirebio Inc.). The circulating biomarker

assays were performed as described by the manufacturer. The methodology of the assay methods has been published previously[4].

For single nucleotide polymorphism (SNP) testing, genomic DNA was extracted from whole blood (Gentra Puregene), according to the manufacturer's protocol. The rs738409 PNPLA3 and rs641738 membrane bound O-acyltransferase domain containing 7 (MBOAT7) SNP were genotyped using TaqMan probe predesigned SNP genotyping assays (Thermo Fisher). Genotyping was performed using StepOne-Plus Real Time PCR System and a Custom TaqMan SNP Genotyping Assay (Applied Biosystems). qPCR reactions were carried out in 10 µl reaction volume containing 4 µl of genomic DNA, 6 µl genotyping master mix with probe.

Statistical evaluation

SPSS version 25.0 (SPSS Inc., Chicago, IL, USA) was used for statistical analyses. Associations between variables were tested using Student's t test, chi-square, or their nonparametric equivalents when appropriate. Bonferroni method was applied to adjust for multiple comparisons. Study period data was divided into three 4-year periods (study period), i.e., 2009–2012; 2013-2016 and 2017–2020. Kaplan-Meier curves and log-rank tests were used to evaluate survival and Cox-regression analysis for the association of survival with sex, age, time period of diagnosis, severity of fibrosis, tumor stage, treatment strategy (variables: curative [surgery, liver transplant or radio frequent ablation], non-curative [TACE or similar and systemic chemotherapy], best supportive care) and liver disease etiology. Multivariable models were developed using the backward likelihood ratio method, which incorporated all significant factors from univariate analysis and the clinically relevant factor: severe fibrosis and cirrhosis. To verify the robustness of the model, it was also tested using the forward stepwise method, with both approaches resulting in the same final model. Analyses were performed in the overall population and in those with non-cirrhotic HCC. All statistical tests were two-sided and evaluated at the 0.05 level of significance.

Supplementary table 1. Characteristics of non-cirrhotic HCC sub-cohort.

		**Cryptogenic HCC**	**NAFLD HCC**	**NAFLD NON-HCC**	**P-value**
**N =**		30	28	18	
**Age**	Median (IQR)	69.5 (16.5)	69.5 (14.3)	62.5 (6)	0.453
**Gender**					0.881
	Female	13 (43%)	13 (47%)	7 (35%)	
	Male	17 (57%)	15 (53%)	11 (65%)	
**Steatosis**		0 (0%)	28 (100%)	28 (100%)	< 0.001
**Diabetes Mellitus**		5 (17%)	13 (47%)	9 (50%)	0.021
**Tumor size**	Median (IQR)	92.5 (59.5)	80 (65)		0.738
**Tumor stage**	Early	7 (20%)	7 (23%)		0.882
	Intermediate	23 (80%)	21 (77%)		
	Advanced	0 (0%)	0 (0%)		
**Tumor characteristics**	Solitary	15 (50%)	18 (60%)		0.392
	Multifocal	14 (47%)	10 (40%)		
	Diffuse	1 (3%)	0 (0%)		

Supplementary table 2. Frequencies of single nucleotide polymorphism

A

p=0.073

**PNPLA3**	**Non-cirrhotic**		
	Cryptogenic-HCC	NAFLD-HCC	Healthy control
**C/C**	19 (57%)	7 (38%)	40 (57%)
**C/G**	9 (27%)	10 (55%)	28 (40%)
**G/G**	5 (15%)	1 (5%)	2 (2%)

B

p=0.122

**MBOAT7**	**Non-cirrhotic**		
	Cryptogenic-HCC	NAFLD-HCC	Healthy control
**C/C**	16 (48%)	4 (22%)	15 (21%)
**C/T**	12 (36%)	7 (38%)	28 (40%)
**T/T**	5 (15%)	7 (38%)	21 (30%)

Table A and B show the frequencies of single nucleotide polymorphism (SNPS) patatin-like phospholipase domain-containing protein 3 (*PNPLA3*) (A) and membrane bound O-acyltransferase domain containing 7 (MBOAT7) (B) in caucasian subjects with non-cirrhotic NAFLD HCC, NAFLD controls and cryptogenic HCC. Associations between variables were tested using the chi-square test.
